# Physicochemical, Rheological and Sensory Evaluation of Herbal Bread Containing Turmeric, Ginger, and Black Cumin Powder

**DOI:** 10.3390/foods13040555

**Published:** 2024-02-12

**Authors:** Muhammad Suffyan Saddique, Muhammad Tauseef Sultan, Shahzad Zafar Iqbal, Christine Bosch, Saeed Akhtar, Hassan Raza, Naima Tariq, Waqas Ahmed

**Affiliations:** 1 Faculty of Food Science and Nutrition, Bahauddin Zakariya University, Multan 60800, Pakistan; suffyanbzu@gmail.com (M.S.S.); saeedakhtar@bzu.edu.pk (S.A.); sh.raza40@yahoo.com (H.R.); naima.tariq799@gmail.com (N.T.); 2 Department of Applied Chemistry, Government College University, Faisalabad 38000, Pakistan; 3 School of Food Science and Nutrition, University of Leeds, Leeds LS2 9JT, UK; c.bosch@leeds.ac.uk; 4 Department of Food Science and Human Nutrition, Faculty of Bio-Sciences, University of Veterinary and Animal Sciences, Lahore 54000, Pakistan; waqas.niaz@uvas.edu.pk

**Keywords:** functional bread, black cumin, turmeric, ginger, rheological behavior, medicinal herbs

## Abstract

The diversity in the global food market is expanding as thousands of new products enter the business every year, among which nutraceutical and functional foods hold important positions. The present research work aimed at the nutritional evaluation of three medicinal herbs, i.e., turmeric (*Curcuma longa* L.), ginger (*Zingiber officinale*), and black cumin (*Nigella sativa*). A bread formulation was enriched with the individual/combined supplementation (1–3%) of these herbs. Later, the bread was analyzed for nutritional, rheological, textural, and sensorial characteristics. The results revealed that the herbs improved the nutritional composition of bread, especially ash and fiber, as the maximum ash and fiber contents were noticed in T_15_ (2.0% dried powder of each plant) with values of 1.64 ± 0.04% and 4.63 ± 0.16%, respectively. The results regarding the rheological behavior showed minor variations in the rheological traits and a slight increase in dough development time up to 4.50 ± 0.20 min in T_10_ from 2.80 ± 0.13 min in T_0_. The sensorial attributes also indicated their marked suitability as external and internal characteristics were least affected by the addition of the herbs. Although some parameters like the crust and crumb colors were affected by the addition of black cumin, showing values of 6.25 ± 0.52 and 4.44 ± 0.19, respectively, in T_15_, and aroma characteristics were affected by the addition of ginger, supplementation with a combination of herbs at lower doses mitigated the adverse effects of other herbs. Moreover, shelf-life extension, especially with the addition of turmeric powder, was the hallmark of this research. This study concluded that medicinal herbs can be incorporated into baked products to improve the nutritional and sensorial attributes of functional herbal bread.

## 1. Introduction

The dawn of the 21st century rejuvenated traditional medicines due to the scientific recognition of the diet–health link. The complete reliance on dietary staples has suffered backlash from nutritionists, and strategies focusing on diet diversification have taken a new leap. As a result, the consumption of natural products has increased significantly, and researchers have attempted to develop novel foods with health-promoting herbs [1]. Most people living in the rural peripheries of developing economies utilize medicinal plants to cure various health disorders. Their knowledge is ancient, and it is feared that the valuable wealth of indigenous knowledge regarding medicinal plants will become unknown to future generations if not documented properly. In the last few years, nutritionists and dietitians started to raise their voices in favor of medicinal plants due to higher costs and side effects associated with synthetic drugs [2,3]. The health-promoting potential of plants is mainly due to the presence of bioactive components in them, and more than eighty thousand metabolites have been characterized [4].

Plants produce ample quantities of primary and secondary metabolites to meet their own requirements. The significant contributions of phytochemicals in dietary regimens improve human health and are linked with the modulation of physiological processes ranging from free radical scavenging to combating various ailments. Some scientists believe that most of the health benefits are due to phytochemicals’ abilities to act as antioxidants and shield the body from the deleterious effects of oxidation and its products, e.g., free radicals. In simple words, antioxidants are necessary to terminate free radical production by modulating chemical reactions and removing intermediates by donating or accepting free electrons [5,6]. This whole scenario has led to the development of various plant-based nutraceuticals and functional foods that have taken hold in the global food market [7,8]. Recently, lifestyle disorders suggested the need for some basic changes in dietary regimens to achieve the health benefits of products. The consumer acceptability of functional products depends, for example, on their similarities with dietary staples. One key strategy for achieving this purpose was to add some functional ingredients in the recipes of some common foods, e.g., bread [9].

Bread is a yeast-leavened product, and historical records suggest it has been consumed since ancient times. It is quite clear from the data that bread was an integral part of Chinese cuisine since pre-historic times (~BC 4000–5000). Although some historical records suggest Europe as its origin, China and Egypt hold rich records of history [10]. In this project, which summarizes the economic importance of bread with respect to its acceptability, emphasis was given to some of the promising plants like ginger (*Zingiber officinale*), black cumin (*Nigella sativa*), and turmeric (*Curcuma longa* L.) as natural health boosters for their incorporation in the development of functional herbal bread. The outcomes of the present research will likely provide scientific insights into functional bread composition and behavior, along with market feasibility and consumer acceptance.

## 2. Materials and Methods

The present research work was performed at the Faculty of Food Science and Technology, Bahauddin Zakariya University, Multan. Three medicinal plants (turmeric, ginger, and black cumin) were investigated.

### 2.1. Acquisition of Raw Material and Preparation of Samples

In the present investigation, medicinal plants were characterized for their suitability in bread development. The procedures followed and materials used are outlined herein. Medicinal plants were purchased from the local market. A representative sample from the sampled population was retained for records in the Faculty of Food Science and Nutrition. Laboratory-grade chemicals were procured for analysis. All necessary items for product development were also purchased from the local market. Medicinal plants, i.e., turmeric, ginger, and black cumin, were air-dried at 105 °C in a hot-air oven, and then the samples were ground with a grinder to obtain a fine powder of the material. Later, sieving was carried out to obtain a powder of uniform particle size. This powder was packed in zipped polythene bags and stored at a refrigeration temperature of 2 °C for future use.

### 2.2. Product Development

Bread was prepared as mentioned in [11] with slight modification at the baking temperature of 220–230 °C for 30–35 min by gradually adding the three best plants selected after nutritional composition profiling of the plants.

### 2.3. Proximate Analysis

The herbal breads containing the herbal powder were assessed for their proximate composition, including moisture, crude protein, crude fat, crude fiber, mineral contents (ash contents), and carbohydrate by difference contents (nitrogen-free extract), following the protocols mentioned in [11].

#### 2.3.1. Rheological Properties

Rheological properties are crucial for studying flow processes, stability, quality control and storage. Wheat flour was blended with the herbal powders as mentioned in Table 1. The rheological behavior was studied at Rahmat Flour Mill, Lahore, using a mixograph. The water absorption, dough development time, dough stability time, weakening index, and Cmax values were recorded using the graph generated by the instrument [11].

#### 2.3.2. Physical Characteristics

The samples were analyzed for bread volume (cm^3^), bread weight, bread density (kg/m^3^), and color (L*) as described in [11]. Bread crust and crumb colors were determined as previously reported [12].

#### 2.3.3. Sensory Evaluation

Breads with different formulations were evaluated for sensory characteristics via descriptive sensory evaluation performed by 20 trained panelists. Before the testing, a Research Consent Form was duly filled out by the trained panelists. The Research Consent Form was for the sensory evaluation of the functional bread. Breads with different formulations were sliced 5 h after baking and placed at room temperature. Slices of bread were placed on disposable plates, and random numbers were assigned to them. Panelists were provided with an ambient environment and water for rinsing before the evaluation of the next sample [13].

### 2.4. Statistical Analysis

Data of the current research were statistically analyzed as previously stated [14]. Statistical software Statistix 8.1 was used by applying completely randomized design (CRD) and two-factor factorial design on the available data. The level of significance was determined through the application of the analysis of variance (ANOVA) technique. Significant difference ranges were measured by using the Duncan multiple range test.

## 3. Results

### 3.1. Proximate Analysis

The statistical analysis (sum of squares) regarding the proximate composition is presented in Table 2. Fat contents exhibited a substantial effect among all the treatments, as depicted by the sum of squares. The results indicated that supplementation with herbal powders at varying concentrations affected the ash and crude fat contents significantly (*p* < 0.05). However, the moisture, protein, crude fiber, and nitrogen-free extract (crude indicator of carbohydrate) varied non-significantly among different treatments. Moisture content is an important indicator of quality as higher moisture results in reduced shelf life. In the present research, moisture contents varied non-significantly among treatments, and moisture content values were in the range of 35.84 ± 1.47 to 37.73 ± 2.38% among all the treatments. The highest moisture content was noted in T_15_ (2.0% powder of each plant), while the lowest moisture content was recorded in bread prepared with T_1_ (0.5% turmeric powder). The protein contents were affected non-significantly due to various levels of herbal powders incorporated in bread, and the mean values depicted clearly that the protein contents of different breads ranged between 9.58 ± 0.36 and 9.84 ± 0.34% (Table 2). The minimum ash contents were found in the control, followed by T_7_ (0.5% black cumin powder). The maximum ash contents were noted in T_15_ (2.0% dried powder of each plant), with the value of 1.64 ± 0.04%, which was statistically on par with T_14_ (1.5% dried powder of each plant), with mean ash contents of 1.60 ± 0.04%. The results exhibited a simple trend; i.e., higher concentrations of plants enhanced the ash contents of bread (Table 2).

Bread is among the bakery products that contain slightly low levels of fat contents; however, fat contents in bread influence some functional properties. The results regarding fat contents revealed a substantial effect of herbal powder supplementation. It is obvious from the means that the minimum fat contents were noticed in the control, whilst the maximum fat contents were recorded for T9 (3.0% black cumin powder), followed by T_15_ (2% powder of each plant). The other treatments like T_1_, T_2_, and T_3_ were statistically on par with each other. Similarly, other treatments like T_5_ and T_6_ and T_12_ and T_13_ were statistically on par with one another. The bread with black cumin powder supplementation exhibited a dose-dependent increase in the fat contents.

The mean values of the fiber contents of the breads supplemented with the powdered herbs are also arranged in Table 2. The minimum value of fiber content was recorded in the control bread, with a value of 4.13 ± 0.34%, whilst the maximum value was noticed in T_15_, with a value of 4.63 ± 0.16%. Although breads with different herbal powders were statistically similar, a higher concentration was recorded in T_15_, which contained 2% powder of each selected plant. The results regarding nitrogen-free extract, representative of carbohydrates in the foods, indicated that carbohydrate contents varied non-significantly among different treatments, with mean values ranging from 43.52 ± 1.23 to 46.43 ± 1.05%.

### 3.2. Rheological Studies

The wheat flour was mixed with the dried powder of herbs according to the treatment plan, and rheological behavior was studied using a mixograph. The results presented in Table 3 indicate that the water absorption capacity of the flour blends varied non-significantly. However, minor variations were recorded, and the highest water absorption was observed in T_10_ (turmeric and ginger, 1.0% each) and T_15_ (2.0% each herb). In contrast, the least water absorption was recorded in the treatment with 1.0% turmeric powder. The variations in the absorption of water may be due to the water-captivation ability of medicinal herbs. This property is directly correlated with the dough development time. The dough development time varied significantly for supplementation with various percentages of black cumin, ginger, turmeric, and their different mixtures, but these variations were observed in narrow ranges. The lowest dough development time was observed in dough supplemented with 2% turmeric. The highest development time was observed in a combined mixture of turmeric and ginger when 5% of this mixture was added to white flour.

The stability of the dough was affected by the spice concentrations. The highest stability was observed in white flour blended with 2% black cumin. Flour blended with a 5% turmeric and ginger mixture showed the lowest stability. Hence, it can be suggested that the stability of flour varies inversely with the DT and WA properties.

The weakening of the dough was altered slightly with the addition of various percentages of spices. Although an increased value of the weakening point was observed in T_10_ (1.0% mixture of turmeric and ginger powder), other herbal combinations resulted in lower values of the weakening index. It can be postulated further that the stability decreased the weakening of the dough taking place. The lowest weakening point was detected in 1.0% black cumin blended flour. The maximum consistency during kneading (Cmax) was also determined in the examination of the rheological properties. The 3% black cumin mixture showed the lowest value, i.e., 432.73 mg/g. Meanwhile, the largest Cmax value (542.27 mg/g) was observed in flour blended with 2% turmeric. This consistency may be due to the various pore sizes of the particles.

### 3.3. Sensory Evaluation: External Characteristics

The external characteristics of bread include loaf volume, crust color, symmetry of form, evenness of bake, and crust characteristics. Slight amendments were made to limit the number of characteristics; e.g., break and shred and crust appearance were described by a cumulative term, crust characteristics. The judges rated the bread on specific performances, with each characteristic being assigned a sensory score. The sensory scores for each trait were analyzed, and the results are tabulated in Table 4. The supplementation of bread with different herbal powders resulted in a marked significant impact on loaf volume and crust color, while symmetry of form, evenness of bake, and crust characteristics were not affected as a function of supplementation.

The means presented in Table 4 indicate that the loaf volume of the bread supplemented with various levels of powdered herbs differed significantly, and the scores ranged between 7.28 ± 0.25 and 8.62 ± 0.37 cm^3^. The minimum loaf volume was recorded in T_15_ with a value of 7.28 ± 0.25 cm^3^, while the maximum was noticed in T_7_ with a value 8.62 ± 0.37 cm^3^. The data also revealed that the loaf volume of control bread (8.51 ± 0.12 cm^3^) was statistically on par with that of T_7_. T_1_, T_4_, T_5_, and T_13_ were statistically on par with each other, with values of 8.28 ± 0.26, 8.27 ± 0.23, 8.24 ± 0.48, and 8.31 ± 0.11 cm^3^, respectively. The loaf volume values were 7.98 ± 0.65, 7.96 ± 0.28, 7.94 ± 0.27, 7.92 ± 0.22 and 7.87 ± 0.43 in T_2_, T_8,_ T_10_, T_12_^,^ and T_14_, respectively. T_3_ and T_9_ also behaved similarly in the said trait. In general, increasing the supplementation level reduced the loaf volume. Amongst spices, black cumin at a lower percentage was rated better in loaf volume. Similarly, the treatments containing equal amounts of the selected spices decreased the sensory score at a higher level.

The supplementation with selected spices at varying levels affected the sensory score for crust color significantly. The results indicated that the judges rated T_0_ (control) the best with the highest sensory score, 8.51 ± 0.12 L*. In general, the sensory score for crust color decreased as a function of increasing concentrations of herbs. The bread with turmeric concentration was rated next to the control with a better score for crust color; however, the yellow color was liked best by some judges. Ginger followed these trends, but its higher supplementation received a lower score for this trait. Among the plants, black cumin affected this trait more than any other plant. The supplementation with 3% black cumin seeds received the second lowest score (6.43 ± 0.33 L*) among all the treatments. The combinations of herbs received a similar score to that of the same herbs alone; e.g., turmeric and ginger supplementation at 1.5% each received a higher score for crust color (8.31 ± 0.58 L*), while the lowest score was recorded for bread with an added mixture of herbs at 2.0% each (6.25 ± 0.52 L*). The other treatments like T_1_, T_4_, T_10_, and T_13_ were statistically on par with each other, and T_2_, T_11_, and T_12_ were also found to behave like each other in the said trait.

The external characteristics like symmetry of form, evenness of bake, and crust characteristics varied non-significantly, and the judges rated almost all breads with slightly similar scores. The mean values regarding the symmetry of form of all the breads prepared with various levels and combinations of herbal powders indicated that the sensory score for the said trait ranged from 4.20 ± 0.18 to 4.57 ± 0.16. The highest values were recorded for T_0_, whereas the lowest were recorded for T_15_ (Table 4). Similar trends were recorded for the evenness of bake and crust characteristics. The sensory scores for these traits varied from 4.41 ± 0.22 to 4.81 ± 0.33 and 4.44 ± 0.19 to 4.75 ± 0.14, respectively. In the evenness of bake, turmeric- and ginger-supplemented bread were rated better, followed by control. In crust characteristics, the control performed better than all the other treatments, and slight variations with lower scores were recorded for the rest of the treatments.

### 3.4. Sensory Evaluation: Internal Characteristics

The internal characteristics of bread include grain, crumb color, aroma, mastication, taste, and texture. As mentioned earlier, the judges were asked to rate the internal characteristics of the bread, and each characteristic was assigned a specific sensory score. The sum of squares (Table 5) indicated that supplementation with herbal powders affected the crumb color, aroma, and taste significantly (*p* < 0.05). In comparison, the grain size, mastication, and texture varied non-significantly among different treatments.

The grain size is an indicator of uniform mixing, phase development, and baking. The supplementation with selected plants at varying levels did not change the grain size significantly, and scores were recorded in the range of 8.03 ± 0.27 to 8.55 ± 0.41 (Table 5). Although the highest score was received by the control, supplementation at lower concentrations received statistically similar scores, e.g., T_1_ (1.0% turmeric powder), T_4_ (1.0% ginger powder), and T_10_ (1% turmeric and 1% ginger powder) with mean scores of 8.55 ± 0.31, 8.53 ± 0.23, and 8.54 ± 0.35, respectively.

The variations in the sensory scores for crumb color followed a similar pattern to that of crust color. The data indicated that all the treatments were substantially different from each other in the said trait (Table 5). The highest values for this trait were recorded for the control (8.50 ± 0.53) bread, while the lowest values (6.13 ± 0.26) were noticed in T_15_ (combination of herbal powders at 2.0% each). Ginger supplementation at 1.0% rated higher in sensory evaluation, and its score, with the value of 8.47 ± 0.51a, was statistically on par with that of T_0_ (control). The other treatments like T_1_, T_2_, T_5_, T_10_, and T_13_ were also statistically on par with each other. Similar to other parameters of sensory evaluation, increasing the concentrations of selected herbs/plants rated slightly lower in the same trait with lowest rating for breads containing black cumin powder.

The mean scores (Table 5) revealed significant differences among treatments regarding aroma. The maximum score of 8.62 ± 0.26 was received by T_0_ (control) for aroma, while the minimum value was recorded for T_15_ (6.40 ± 0.22). Treatments like T_1_, T_7_, T_8_, T_11_, and T_13_ were statistically on par with each other, with mean scores of 8.37 ± 0.33, 8.42 ± 0.38, 8.24 ± 0.39, 8.50 ± 0.26, and 8.18 ± 0.25, respectively. Bread supplemented with black cumin powder received higher scores for aroma, followed by turmeric-supplemented bread. The lowest scores for aroma were recorded for ginger; however, co-supplementation with ginger and black cumin at 1.0% each masked the aroma of ginger significantly (8.50 ± 0.26). The higher concentrations of supplementation, e.g., T_15_ (combination of all herbs at 2.0% each), received the lowest scores from the trained taste panelists.

Taste is the second most important sensory characteristic after physical appearance, and it was affected significantly by the types of herbs, their varying levels, and their combinations. The judges rated the control with the highest score (8.32 ± 0.28), while the lowest score (6.47 ± 0.25) for the said trait was recorded for T_15_ (combination of all plants at 2.0 each). The other treatments like T_1_, T_2_, T_7_, T_8_, T_11_, and T_12_ were statistically on par with each other with the values of 8.17 ± 0.27, 7.86 ± 0.16, 8.23 ± 0.25, 8.07 ± 0.45, 8.21 ± 0.36, and 8.13 ± 0.35, respectively. Similarly, T_3_ (3.0 turmeric powder), T_5_ (2.0% ginger powder), and T_13_ (turmeric and black cumin at 1.0% each) were also found to behave like each other. Interestingly, T_11_ (ginger and black cumin at 1.0 each) was rated better in the sensory score for taste, with a mean score of 8.21 ± 0.36. However, the combination of all three herbs was rated on the lower side.

Mastication is a characteristic that is linked to the mixing of saliva with food in the oral cavity and also includes chewing behavior. In the present study, supplementation did not affect this trait momentously (Table 5). The scores for mastication were in the range of 7.91 ± 0.30 to 8.53 ± 0.39. However, the control showed fairly good mastication as compared to the rest of the treatments. Like previous parameters, a declining trend was observed for the supplementation level. Like mastication, non-significant differences were recorded for the texture of bread, and overall values ranged between 12.38 ± 0.55 and 13.61 ± 0.60. The highest values were recorded for T_0_, whereas the lowest were recorded for T_1_. The judges’ responses were a bit varying in this trait, and nearly all the bread was rated in the same range of 11.50 to 13.50 (at the level of replication), reflecting the similar textural properties of breads prepared with varying levels of herbs and their combinations.

### 3.5. Sensory Evaluation: Cumulative Internal, External, and Total Scores

The external and internal characteristics are discussed in depth in the last section; however, the cumulative internal, external, and total scores are important as they reflect the overall acceptability of the products. The results showing the means for total external score (Table 6) revealed a significant variation, but as far as commercial baking experiences are concerned, the scores are still in ranges considered widely acceptable by the trained taste panelists. The major bottleneck for variations was crust color, where treatments containing black cumin were rated lower, and a similar trend was replicated in the total external score as well. The mean scores indicated that T_0_ (control) received the maximum external score (31.02 ± 034) and was statistically on par with T_4_ (1.0% ginger powder), T_10_ (turmeric and ginger powder at 1.0 each), and T_13_ (combination of herbal powders at 1.0 each) with mean scores of 30.89 ± 0.61, 30.79 ± 0.41, and 30.82 ± 0.55, respectively. As mentioned earlier, the bread containing higher levels of black cumin powder received lower scores, e.g., T_9_ (3.0% black cumin powder) and T_15_ (combination of herbs at 1.0% each) with mean scores of 27.29 ± 0.61 and 27.00 ± 0.74, respectively.

The results for internal scores differed from those fo external scores due to significant variations in crumb color, aroma, and taste. The total internal score (Table 6) revealed significant variations, with T_0_ (control) receiving the maximum internal score (56.13 ± 1.11). In comparison, the lowest score (47.68 ± 0.48) was received by T_15_ (combination of herbs at 2.0% each). Bread supplemented with black cumin powder was rated better for internal parameters, followed by bread supplemented with turmeric, and the lowest internal scores were awarded to ginger-supplemented bread. Among the combination of herbs, bread supplemented with ginger and black cumin was rated highest, with a mean internal score of 54.91 ± 0.94. Interestingly, black cumin masked the aroma and taste of ginger significantly, and thus judges rated this combination higher on sensory scores.

The total cumulative score represents the overall performance of treatments. In the present research, T_0_ (control) received the highest cumulative score (87.15 ± 1.40), followed by T_11_ (ginger and black cumin powder at 1.0% each) with a mean score of 84.42 ± 2.00. In general, breads prepared with low concentrations of herbs (1.0%) were rated higher by the judges; e.g., T_1_, T_4_, T_7_, and T_13_ behaved alike with mean scores of 83.74 ± 0.42, 83.30 ± 0.94, 82.98 ± 1.43, and 83.21 ± 0.71, respectively. Overall, the results from the sensory evaluation are quite promising, with significant commercial importance.

## 4. Discussion

Consumer awareness is increasing due to easy access to knowledge sources, especially for consumers living in the modern-day world, e.g., urban areas as well as urban peripheries. The science of nutrition, which has grown at an unprecedented pace in the last few decades, has highlighted the negative impact of dietary patterns [15]. The addition of functional ingredients is gaining a wide range of consumer acceptability but the selection of functional ingredients due to social and religious acceptance is a matter of great concern. Bread is consumed in all cultures by all age groups and thus holds a prominent place in dietary patterns. Bread usually prepared from white flour or straight-grade flour results in the loss of components present in the bran portion. Researchers across the globe have paid attention to improving the quality of bread, focusing on ingredients, functional properties, and process improvement [16]. The quality of ingredients is the fundamental part of the whole process, and it can be considered a crux of research as well. Quality flour contains appreciable quantities (~10–12%) of gluten, especially high-molecular-weight fractions of glutenin. The sub-fractions of gluten and the gliadin-to-glutenin ratio also impact the viscoelasticity of bread dough [17,18]. Starch accounts for more than 75% of total wheat and is thus equally important in ensuring the quality of bread. The quality of starch, with special reference to the amylose/amylopectin ratio, is very important in the viscoelastic dough network and oven spring. The particle size of flour and starch can be ranked third in its significance for improving the quality of bread [19]. Although many other factors are also important, their discussion would be too lengthy. It is therefore imperative to just highlight these factors, i.e., wheat type (hard or soft), season (summer, spring, and winter wheat), milling quality, extraction rate, flour particle size, and damaged starch and non-starch polysaccharides [20]. The rheological factors include viscosity, water absorption capacity, mixing tolerance, dough development time, falling number, and amylo-graphic properties. Rheological characteristics are also dependent on quality factors, especially on the composition of wheat flour mentioned above [21,22]. However, their assessment is vital in designing the appropriate processing conditions. The recipe and formulation of ingredients require complete knowledge of bread development and their potential impacts on the quality of finished produce. The list of ingredients and their effects is too large to discuss in this section as complete books are available on this topic. However, yeast, milk powder, salt, sugar (especially sucrose), bread improvers, preservatives are of key importance [23]. The bread improver is blend of various components including oxidizing/reducing agents, enzymes, self-raising agents, color, flavors, taste improver etc. The processing also impacts the quality as mixing machines, their spirals, mixing speed, fermentation time, proofing time, proofing conditions, baking temperature, oven type, cooling time and temperature conditions. The storage conditions (temperature, humidity and time) of the bread also affect the quality of finished products for end consumers [24,25]. In the present research, recipe was optimized, and such effects were confounded accordingly.

In the recent past, significant research has been conducted to add dietary fiber especially soluble dietary fiber but limited research has been conducted to add some antioxidant sources [26,27]. In a study, corn bran fibre bread had lower dietary fiber contents of 3.79 and 3.98% compared to the control sample (4.56%). This might probably be due to interaction between the fiber components of corn bran and other dough ingredients. It was observed in a similar study that coconut enriched bread with dietary fiber of 5.28% can deliver 21.12% of daily requirements of dietary fiber. The crude protein content of the bread samples ranged from 4.56 to 5.15% [28]. Dietary fiber addition presents some problems as well due to its interaction with gluten network impacting the bread volume. However, such problems can be addressed by slightly increasing the levels of bread improver, more stay time and indeed milling and grinding of fiber particles that would be limiting their negative impacts. The dietary fiber of plants/herbs added, using composite flour technology received attention of researchers and significant number of articles are available in the literature. However, impact of those ingredients on quality improvement and health impact is limited. Pakistan is rich in flora and fauna but least research has been conducted on the development of functional bread usually indigenous resources [29]. The social development and economic profile of communities varies in different part of Pakistan. The regions like South Punjab lack far behind than urban epicenters like Lahore and Karachi [30].

In the present research efforts were made to use plants commonly known to the communities thus products made from them could be commercially viable. For this purpose, the plants used in cuisine like turmeric and ginger were selected. The plants like bitter apple, Senna and black cumin are used by rural communities to prevent and cure various health maladies. In the present research, addition of turmeric did not affect the proximate composition of bread as depicted in the results; however, it slightly changed the color tone of the bread especially at higher ratio. The sensory experts adjudged that turmeric can be added at higher rates for example up to 3 to 5%. The same comments are validated by some scientists and reported that that turmeric of 4% can be used in functional bread. However, our research group is of view that commercialization and long-term prospects of the herbal products requires lower levels of turmeric, i.e., 1 to 3% that will make it commercially viable [31,32]. The addition of ginger did not affect the proximate and color composition of bread significantly, however its incorporation at higher rates affected the aroma profile of the bread. The sensory judges highlighted the issue of undesirable pungency that might be due to higher temperature of the baking. The temperature of baking is usually 220–240 °C that increased the temperature of bread dough to 200 °C outer side (Crust of the bread) and 120 °C for inner, i.e., crumb of bread [33]. The temperature differences inside and outside of bread dough result in crunchy crust with mild ginger flavor. In comparison, the inner crumb remains soft and dumb with significant ginger flavor. It has been highlighted in many research trials that temperature changes the volatile composition of the ginger due to inter-conversion of bioactive components of the ginger especially gingerols and shagols [34,35].

Moisture content is a key parameter which is used to determine bread shelf-stability and susceptibility to microbial infections. Decrease in the moisture of freshly baked bread was observed. The water contents present in the crumb usually migrate to crust as well as slightly evaporate thus moisture contents usually decline with the passage of time. The crumb of freshly baked bread contained about 47% moisture. However, during 2 h of cooling the moisture dropped to 41%. A study reported that the moisture content of calcium enriched bread decreased by 9% in 3 days of initial storage [36]. The ash content in control bread was 0.79% but with the addition of turmeric, this quantity was increased. This anomalous increase of ash content of the product is dependent on the ash content of the turmeric powder that was about four times higher than that of wheat flour [37]. Black cumin addition due to its black tonality changed the color of bread from white to slightly blackish tinge in the crumb of the bread. Rest of the parameters were least affected by the addition. The sensory judges of the present research adjudged the lower doses more suitable for all groups. Interestingly, the consumer’s dietary patterns are influenced by social and religious perception and judges were of the view that it will be accepted by masses due to religious interest of the Muslims. The addition of the herbal mixtures affects in quite different way as ginger aroma was masked by the addition of black cumin and turmeric. Similarly, the bittering tinge of the turmeric was also least noticed when combinations of the herbs were used [38,39]. The data revealed that the treatments imparted non-significant effect on the moisture, protein, fat, and carbohydrate contents of the bread. However, statistically significant differences were recorded in ash and fiber contents of the bread. The maximum levels of ash and fiber were recorded in bread with herbal decoction containing 1.5% of each herb, i.e., turmeric, ginger and black cumin. The sensorial portrayal of the research as highlighted in the last paragraph depicted some interesting results [40]. The sensory characteristics like symmetry of form, grain size, mastication and texture varied from each but statistically all treatments were on par with each other. The addition of herbs and their blends affected the loaf volume and minimum score of 7.28 ± 0.25 was observed for T_15_ (herbs at 2% each), while the maximum was noticed in T_7_ (8.62 ± 0.37). There was found a significant difference of crust color among all treatments. The highest crust and crumb color scores were recorded for T_0_ (8.51 ± 0.44), while the lowest for the said trait were found in T_15_ (6.25 ± 0.52). Similar trends were observed for the aroma scores and maximum score was observed for T_0_ (8.62 ± 0.26), while the minimum values were recorded in T_15_, i.e., 6.40 ± 0.22. Likewise, sensory panel gave the highest taste score to control bread (8.32 ± 0.28), while the lowest to T_15_, i.e., 6.47 ± 0.25. However, in all last three parameters discussed, the products containing 1% herbs and combination of 1.0% of herb each was granted the status of commercially acceptable. The results also support the past research [38,39,41]. Amongst sensory traits, acceptable color tonality is an inevitable quality trait because the product color usually influences the consumer acceptability. The color as mentioned early is affected by ingredients, processing factors and shelf-life of the produced bread is evident from the previous works [42]. It was reported that the baking conditions affect the color of the crust technically [43]. As the bread formulation remains the same in all the samples, it can be assumed that the crumb color characteristics are not liable to differ significantly [44]. Similarly, loaf volume characteristic is liked by processors and consumers and different scientists studied the effects of herbal ingredients on the volume of bread. In one study, fennel seed was assessed for its incorporation and results showed a significant decrease (*p* > 0.05). Gluten network impairment due to wheat flour substitution and interaction between gluten and fiber led to lowering CO_2_ retention capacity of dough that could be the major reasons for the decreased loaf volume [45] as well as the proofing time and temperature [46].

## 5. Conclusions

The present research revealed that the medicinal herbs improved the nutritional composition of bread especially ash and fiber contents. Maximum levels of ash and fiber were recorded in bread with herbal decoction containing 1.5% of each herb. The sensory attributes also indicated their marked suitability as external and internal characteristics were least affected by the addition of the herbs. The shelf life extension especially with the addition of the herbal powder was the hall mark of the research. The study concluded that medicinal herbs can be incorporated in baked goods to contribute nutritional and textural qualities.

## Figures and Tables

**Table 1:** Treatments used in the study (per 100 g dough).

Treatments	Turmeric (%)	Ginger (%)	Black Cumin (%)
T_0_	-	-	-
T_1_	1.00	-	-
T_2_	2.00	-	-
T_3_	3.00	-	-
T_4_	-	1.00	-
T_5_	-	2.00	-
T_6_	-	3.00	-
T_7_	-	-	1.00
T_8_	-	-	2.00
T_9_	-	-	3.00
T_10_	1.5	1.5	-
T_11_	-	1.5	1.5
T_12_	1.5	-	1.5
T_13_	1	1	1
T_14_	1.5	1.5	1.5
T_15_	2	2	2

**Table 2:** Effect of herbal extracts on proximate composition (%) of bread.

Treatment	Moisture	Ash	Protein	Fat	Fiber	CHO
T_0_	36.15 ± 0.42	1.45 ± 0.04^e^	9.60 ± 0.34	2.24 ± 0.11^h^	4.13 ± 0.34	46.43 ± 1.05
T_1_	35.84 ± 1.47	1.49 ± 0.05^c–e^	9.67 ± 0.17	2.30 ± 0.08^f–h^	4.27 ± 0.16	46.43 ± 2.59
T_2_	36.45 ± 0.26	1.52 ± 0.05^c–e^	9.62 ± 0.65	2.33 ± 0.10^e–h^	4.36 ± 0.14	45.72 ± 1.40
T_3_	37.12 ± 1.69	1.55 ± 0.05^b–d^	9.61 ± 0.50	2.37 ± 0.01^d–h^	4.47 ± 0.17	44.88 ± 1.15
T_4_	36.14 ± 1.42	1.48 ± 0.01^de^	9.58 ± 0.36	2.27 ± 0.13^gh^	4.23 ± 0.19	46.30 ± 1.02
T_5_	36.22 ± 2.38	1.52 ± 0.05^cde^	9.59 ± 0.44	2.30 ± 0.02^f–h^	4.34 ± 0.18	46.03 ± 1.74
T_6_	36.52 ± 1.29	1.56 ± 0.02^bc^	9.61 ± 0.29	2.34 ± 0.04^e–h^	4.46 ± 0.17	45.51 ± 1.39
T_7_	36.18 ± 2.16	1.48 ± 0.03^de^	9.66 ± 0.41	2.44 ± 0.10^d–g^	4.17 ± 0.12	46.07 ± 2.43
T_8_	36.23 ± 0.52	1.50 ± 0.03^cde^	9.74 ± 0.43	2.64 ± 0.15^bc^	4.21 ± 0.18	45.68 ± 1.63
T_9_	36.45 ± 1.06	1.53 ± 0.04^b–d^	9.84 ± 0.34	2.84 ± 0.08^a^	4.27 ± 0.10	45.07 ± 0.79
T_10_	36.21 ± 1.35	1.52 ± 0.06^c–e^	9.59 ± 0.52	2.32 ± 0.07^e–h^	4.34 ± 0.17	46.02 ± 2.28
T_11_	36.20 ± 0.60	1.51 ± 0.06^c–e^	9.67 ± 0.13	2.47 ± 0.12^d–f^	4.28 ± 0.22	45.87 ± 1.15
T_12_	36.48 ± 1.57	1.52 ± 0.02^c–e^	9.70 ± 0.38	2.49 ± 0.09^c–e^	4.30 ± 0.04	45.51 ± 1.79
T_13_	37.21 ± 0.95	1.55 ± 0.01^b–d^	9.67 ± 0.30	2.52 ± 0.02^cd^	4.39 ± 0.23	44.66 ± 1.74
T_14_	37.52 ± 1.53	1.60 ± 0.04^ab^	9.70 ± 0.41	2.65 ± 0.02^bc^	4.52 ± 0.28	44.01 ± 1.07
T_15_	37.73 ± 2.38	1.64 ± 0.04^a^	9.70 ± 0.30	2.78 ± 0.14^ab^	4.63 ± 0.16	43.52 ± 1.23
Sum of squares	13.610 ^ns^	0.100 **	0.211 ^ns^	1.545 **	0.803 ^ns^	32.627 ^ns^

Data: means ± SD. Same letters in columns show non-significant differences among treatments. ^ns^ = non-significant, ** = significant.

**Table 3:** Effect of herbal powder on rheological characteristics of wheat–herbal powder blends.

Treatment	Water Absorption %	Dough Development Time (Min)	Dough Stability Time (Min)	Weakening Index (BU)	Cmax (mg/g)
T_0_	58.40 ± 2.18	2.80 ± 0.13^f^	10.04 ± 0.15^h^	32.54 ± 1.85^b^	532.29 ± 27.26^ab^
T_1_	58.00 ± 1.62	2.50 ± 0.17^g^	9.50 ± 0.39^i^	29.00 ± 0.33^c^	520.91 ± 36.75^b^
T_2_	58.20 ± 2.14	2.00 ± 0.06^h^	11.00 ± 0.45^f^	35.00 ± 1.02^a^	542.27 ± 22.60^a^
T_3_	58.30 ± 1.16	2.50 ± 0.02^g^	12.00 ± 0.08^d^	27.00 ± 0.91^de^	534.09 ± 7.23^ab^
T_4_	58.28 ± 1.84	3.25 ± 0.17^e^	10.40 ± 0.37^gh^	28.00 ± 1.33^cd^	493.00 ± 23.21^cd^
T_5_	58.39 ± 2.07	3.35 ± 0.08^de^	10.50 ± 0.26^g^	29.00 ± 1.64^c^	501.60 ± 22.83^c^
T_6_	58.42 ± 2.31	3.45 ± 0.07^cd^	10.20 ± 0.57^gh^	32.00 ± 0.78^b^	489.09 ± 17.82^cde^
T_7_	58.90 ± 1.98	3.50 ± 0.23^c^	12.50 ± 0.31^c^	20.00 ± 0.25^h^	444.55 ± 14.63^gh^
T_8_	58.90 ± 3.42	3.50 ± 0.11^c^	14.50 ± 0.84^b^	23.00 ± 0.99^fg^	446.36 ± 21.44^gh^
T_9_	58.70 ± 4.18	4.00 ± 0.13^b^	16.50 ± 0.71^a^	23.00 ± 0.93^fg^	432.73 ± 8.69^h^
T_10_	59.60 ± 0.48	4.50 ± 0.20^a^	8.50 ± 0.16^j^	32.00 ± 1.08^b^	488.64 ± 13.30^cde^
T_11_	59.00 ± 1.98	4.00 ± 0.11^b^	10.50 ± 0.42^g^	26.00 ± 1.16^e^	462.27 ± 13.47^fg^
T_12_	59.10 ± 1.28	3.50 ± 0.20^c^	11.50 ± 0.26^e^	24.00 ± 0.44^f^	466.36 ± 16.04^f^
T_13_	59.10 ± 3.11	3.50 ± 0.02^c^	9.50 ± 0.26^i^	28.00 ± 1.13^cd^	460.45 ± 25.07^fg^
T_14_	59.20 ± 0.95	3.50 ± 0.13^c^	11.00 ± 0.36^f^	22.00 ± 0.94^g^	473.64 ± 13.08^ef^
T_15_	59.20 ± 1.24	3.50 ± 0.10^c^	10.50 ± 0.62^g^	24.00 ± 0.35^f^	476.36 ± 16.67^def^
Sum of squares	9.38 ^ns^	17.67 **	176.96 **	837.87 **	51347.02 **

Data: means ± SD. Same letters in columns show non-significant differences among treatments. ^ns^ = non-significant, ** = significant.

**Table 4:** Effect of herbal powder on external characteristics of bread.

Treatment	Loaf Volume (cm^3^)	Crust Color (L*)	Symmetry of Form	Evenness of Bake	Crust Characteristics
T_0_	8.51 ± 0.12^a^	8.51 ± 0.44^a^	4.57 ± 0.16	4.68 ± 0.45	4.75 ± 0.14
T_1_	8.28 ± 0.26^a–c^	8.28 ± 0.44^a^	4.25 ± 0.07	4.43 ± 0.38	4.62 ± 0.18
T_2_	7.98 ± 0.65^b–d^	7.98 ± 0.51^ab^	4.27 ± 0.16	4.63 ± 0.28	4.48 ± 0.36
T_3_	7.50 ± 0.28^de^	7.69 ± 0.39^d^	4.34 ± 0.22	4.46 ± 0.37	4.65 ± 0.22
T_4_	8.27 ± 0.23^a–c^	8.27 ± 0.22^a^	4.30 ± 0.36	4.56 ± 0.26	4.74 ± 0.15
T_5_	8.24 ± 0.48^a–c^	7.64 ± 0.43^bc^	4.49 ± 0.22	4.61 ± 0.15	4.64 ± 0.11
T_6_	7.81 ± 0.49^cd^	7.41 ± 0.33^b–d^	4.23 ± 0.06	4.46 ± 0.21	4.54 ± 0.19
T_7_	8.62 ± 0.37^a^	7.62 ± 0.32^bc^	4.28 ± 0.20	4.51 ± 0.24	4.66 ± 0.23
T_8_	7.96 ± 0.28^b–d^	6.96 ± 0.46^de^	4.54 ± 0.15	4.67 ± 0.13	4.55 ± 0.19
T_9_	7.62 ± 0.27^de^	6.43 ± 0.33^ef^	4.55 ± 0.32	4.47 ± 0.18	4.55 ± 0.13
T_10_	7.94 ± 0.27^b–d^	8.36 ± 0.36^a^	4.22 ± 0.22	4.81 ± 0.33	4.57 ± 0.23
T_11_	8.36 ± 0.38^ab^	7.94 ± 0.36^ab^	4.47 ± 0.19	4.45 ± 0.30	4.44 ± 0.19
T_12_	7.92 ± 0.22^b–d^	7.92 ± 0.38^ab^	4.35 ± 0.24	4.69 ± 0.37	4.65 ± 0.26
T_13_	8.31 ± 0.11^a–c^	8.31 ± 0.58^a^	4.30 ± 0.46	4.49 ± 0.10	4.70 ± 0.20
T_14_	7.87 ± 0.43^b–d^	7.27 ± 0.51^cd^	4.38 ± 0.25	4.42 ± 0.19	4.67 ± 0.35
T_15_	7.28 ± 0.25^e^	6.25 ± 0.52^f^	4.20 ± 0.18	4.41 ± 0.22	4.58 ± 0.22
Sum of squares	10.212 **	36.366 **	1.192 ^ns^	1.087 ^ns^	0.577 ^ns^

Data: means ± SD. Same letters in columns show non-significant differences among treatments. ^ns^ = non-significant, ** = significant.

**Table 5:** Effect of herbal powder on internal characteristics of bread.

Treatment	Grain	Crumb Color (L*)	Aroma	Mastication	Taste	Texture
T_0_	8.55 ± 0.41	8.50 ± 0.53^a^	8.62 ± 0.26^a^	8.53 ± 0.39	8.32 ± 0.28^a^	13.61 ± 0.60
T_1_	8.55 ± 0.31	8.16 ± 0.39^a–c^	8.37 ± 0.33^a–c^	8.53 ± 0.34	8.17 ± 0.27^a–c^	12.38 ± 0.55
T_2_	8.48 ± 0.21	8.06 ± 0.35^a–d^	8.12 ± 0.35^b–d^	8.12 ± 0.66	7.86 ± 0.16^a–e^	12.97 ± 0.67
T_3_	8.29 ± 0.27	7.27 ± 0.21^ef^	7.24 ± 0.23^g^	8.14 ± 0.21	7.46 ± 0.24^ef^	13.05 ± 0.80
T_4_	8.53 ± 0.23	8.47 ± 0.51^a^	7.53 ± 0.31^e–g^	7.98 ± 0.21	7.73 ± 0.26^c–f^	13.16 ± 0.56
T_5_	8.41 ± 0.66	7.85 ± 0.30^b–d^	6.44 ± 0.33^h^	8.37 ± 0.38	7.28 ± 0.36^f^	12.88 ± 1.66
T_6_	8.30 ± 0.35	7.27 ± 0.56^ef^	6.35 ± 0.18^h^	8.13 ± 0.26	6.79 ± 0.38^g^	12.70 ± 0.40
T_7_	8.37 ± 0.61	7.62 ± 0.54^de^	8.42 ± 0.38^a–c^	8.21 ± 0.43	8.23 ± 0.25^ab^	12.92 ± 0.51
T_8_	8.28 ± 0.27	7.12 ± 0.23^ef^	8.24 ± 0.39^a–d^	8.60 ± 0.48	8.07 ± 0.45^a–d^	13.23 ± 0.70
T_9_	8.14 ± 0.27	6.75 ± 0.45^f^	7.80 ± 0.51^d–f^	8.16 ± 0.26	7.63 ± 0.23^d–f^	12.82 ± 0.62
T_10_	8.54 ± 0.35	7.80 ± 0.18^b–d^	8.01 ± 0.10^cd^	8.22 ± 0.32	7.83 ± 0.38^b–e^	12.64 ± 0.44
T_11_	8.46 ± 0.26	8.30 ± 0.47^ab^	8.50 ± 0.26^ab^	8.57 ± 0.34	8.21 ± 0.36^ab^	12.87 ± 0.89
T_12_	8.22 ± 0.68	7.65 ± 0.25^c–e^	7.90 ± 0.38^de^	8.24 ± 0.35	8.13 ± 0.35^a–c^	12.86 ± 0.79
T_13_	8.54 ± 0.46	8.21 ± 0.08^ab^	8.18 ± 0.25^a–d^	7.91 ± 0.30	7.32 ± 0.34^f^	13.24 ± 0.51
T_14_	8.12 ± 0.24	7.20 ± 0.27^ef^	7.44 ± 0.29^fg^	8.02 ± 0.38	6.87 ± 0.45^g^	12.65 ± 0.39
T_15_	8.03 ± 0.27	6.13 ± 0.26^g^	6.40 ± 0.22^h^	8.26 ± 0.20	6.47 ± 0.25^g^	12.39 ± 0.31
Sum of squares	2.211 ^ns^	32.579 **	43.497 **	3.459 ^ns^	24.408 **	7.667 ^ns^

Data: means ± SD. Same letters in columns show non-significant differences among treatments. ^ns^ = non-significant, ** = significant.

**Table 6:** Effect of herbal powder on internal, external, and total scores of bread.

Treatment	External Score	Internal Score	Total Score
T_0_	31.02 ± 0.34^a^	56.13 ± 1.11^a^	87.15 ± 1.40^a^
T_1_	30.27 ± 0.66^a–d^	54.16 ± 0.45^bc^	83.74 ± 0.42^b^
T_2_	29.91 ± 0.68^de^	53.61 ± 1.64^bc^	82.67 ± 2.86^bc^
T_3_	28.56 ± 0.32^g^	51.45 ± 0.66^de^	78.98 ± 0.48^de^
T_4_	30.89 ± 0.61^ab^	53.40 ± 0.67^bc^	83.30 ± 0.94^b^
T_5_	30.06 ± 0.39^b–e^	51.23 ± 3.20^ef^	80.49 ± 4.39^cd^
T_6_	29.30 ± 0.30^e–g^	49.54 ± 0.41^f^	77.61 ± 0.80^e^
T_7_	29.84 ± 0.41^de^	53.77 ± 0.83^bc^	82.98 ± 1.43^bc^
T_8_	28.78 ± 0.70^fg^	53.54 ± 1.26^bc^	82.04 ± 1.32^bc^
T_9_	27.29 ± 0.61^h^	51.30 ± 0.67^d–f^	78.67 ± 0.62^de^
T_10_	30.79 ± 0.41^ab^	53.04 ± 1.23^b–d^	82.54 ± 1.35^bc^
T_11_	29.71 ± 0.68^de^	54.91 ± 0.94^ab^	84.42 ± 2.00^b^
T_12_	29.99 ± 1.12^c–e^	53.00 ± 2.02^cd^	82.07 ± 2.77^bc^
T_13_	30.82 ± 0.55^ab^	53.40 ± 0.68^bc^	83.21 ± 0.71^b^
T_14_	29.48 ± 0.80^d–f^	50.30 ± 0.90^ef^	78.61 ± 0.99^de^
T_15_	27.00 ± 0.74^h^	47.68 ± 0.48^g^	74.06 ± 0.27^f^
Sum of squares	344.012 **	107.590 **	754.855 **

Data: means ± SD. Same letters in columns show non-significant differences among treatments. ** = significant.

## Data Availability

Data in this study are available upon request from corresponding authors.
